# Harmonizing solubility measurement to lower inter-laboratory variance – progress of consortium of biopharmaceutical tools (CoBiTo) in Japan

**DOI:** 10.5599/admet.704

**Published:** 2019-08-05

**Authors:** Asami Ono, Naoya Matsumura, Takahiro Kimoto, Yoshiyuki Akiyama, Satoko Funaki, Naomi Tamura, Shun Hayashi, Yukiko Kojima, Masahiro Fushimi, Hiroshi Sudaki, Risa Aihara, Yuka Haruna, Maiko Jiko, Masaru Iwasaki, Takuya Fujita, Kiyohiko Sugano

**Affiliations:** 1 Asahi Kasei Pharma Corporation, 632-1 Mifuku, Izunokuni, Shizuoka 410-2321, Japan; 2 Ono Pharmaceutical Co., Ltd., 3-3-1, Sakurai, Shimamoto-cho, Mishima-gun,Osaka 618-8585, Japan; 3 Central Pharmaceutical Research Institute, Japan Tobacco Inc, 1-1 Murasaki-cho, Takatsuki, Osaka 569-1125, Japan; 4 Shionogi & Co., Ltd., 3-1-1, Futaba-cho, Toyonaka-shi,Osaka 561-0825, Japan; 5 Sumitomo Dainippon Pharma Co., Ltd., 3-1-98 Kasugadenaka, Konohana-ku, Osaka 554-0022, Japan; 6 Sawai Pharmaceutical Co., Ltd., 5-2-30, Miyahara, Yodogawa-ku, Osaka 532-0003, Japan; 7 Nippon Boehringer Ingelheim Co. Ltd., 6-7-5 Minatojima-minamimachi, Chuo-ku, Kobe, Hyogo 650-0047, Japan; 8 Towa Pharmaceutical Co., Ltd., 134 Chudoji Minami-machi, Shimogyo-ku, Kyoto 600-8813, Japan; 9 Daiichi Sankyo RD Novare Co., Ltd, 1-16-13 Kitakasai, Edogawa-ku, Tokyo 134-8630, Japan; 10 College of Pharmaceutical Sciences, Ritsumeikan University, 1-1-1 Noji-higashi, Kusatsu, Shiga 525-8577, Japan

**Keywords:** shake-flask solubility, equilibrium solubility, poorly water-soluble drugs

## Abstract

The purpose of the present study was to harmonize the protocol of equilibrium solubility measurements for poorly water-soluble drugs to lower inter-laboratory variance. The “mandatory” and “recommended” procedures for the shake-flask method were harmonized based on the knowledge and experiences of each company and information from the literature. The solubility of model drugs was measured by the harmonized protocol (HP) and the non-harmonized proprietary protocol of each company (nonHP). Albendazole, griseofulvin, dipyridamole, and glibenclamide were used as model drugs. When using the nonHP, the solubility values showed large inter-laboratory variance. In contrast, inter-laboratory variance was markedly reduced when using the HP.

## Introduction

In recent years, drug candidates have tended to be poorly water-soluble [1-3]. The oral absorption of a drug is determined by the solubility, dissolution rate, and membrane permeability of the drug in the gastrointestinal tract [4-7]. Poor water-solubility often causes poor and variable oral absorption [8-11]. Therefore, solubility measurements are routinely performed in drug discovery and development [12-15]. The quality of solubility data is critically important for drug design [16-19], oral absorption prediction [20-23], and formulation development [24-26]. However, the solubility values of poorly water-soluble drugs vary depending on the experimental procedures [27].

Recently, Avdeef et al. reported consensus recommendations on the experimental procedures of equilibrium solubility measurements by the shake-flask method [28]. They discussed various aspects of equilibrium solubility measurements, such as incubation temperature, incubation time, the separation method of solids from solution, and pH measurements.

As a part of the Consortium of Biopharmaceutical Tools (CoBiTo) project, we conducted a questionnaire concerning the solubility measurements of each company. It was revealed that each company employs different experimental procedures (Table 1), suggesting that there would be large inter-laboratory variance in solubility data.

**Figure 1.**
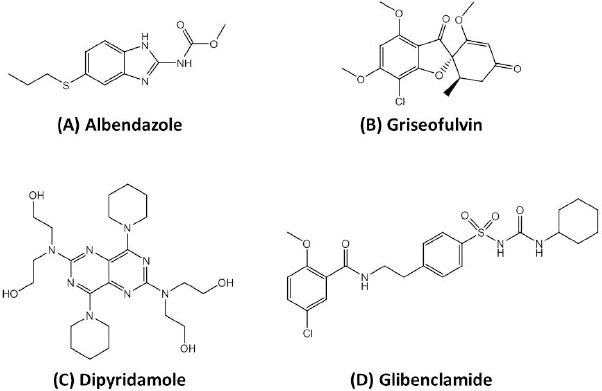
Chemical structures of model drugs.

**Figure 2.**
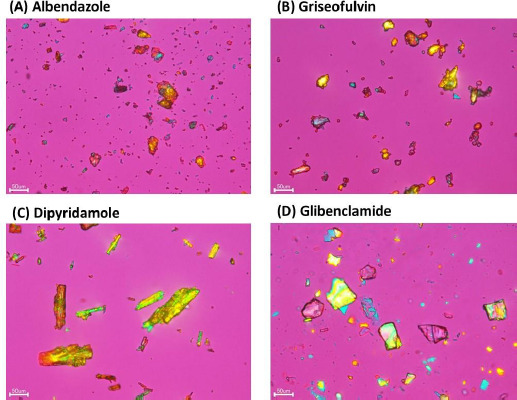
Polarized light microscopy images of model drugs (ECLIPSE Ti (Nikon, Japan)).

The purpose of the present study was to harmonize the experimental procedures of solubility measurements by the shake-flask method for low solubility drugs to lower inter-laboratory variance. Albendazole, griseofulvin, dipyridamole, and glibenclamide were used as model drugs (Table 2, Figures 1 and 2).

## Experimental

### Materials

Albendazole was purchased from Tokyo Chemical Industry Co., Ltd (Tokyo, Japan). Griseofulvin, dipyridamole, and glibenclamide were purchased from FUJIFILM Wako Pure Chemical Corporation (Osaka, Japan). An identical batch was used by all companies.

The Japanese Pharmacopeia (JP) 2^nd^ fluid for dissolution testing (12.5 mM KH_2_PO_4_ and 12.5 mM Na_2_HPO_4_, pH 6.8, buffer capacity 10 mM/ ΔpH) was used for the solubility measurement.

### Equilibrium solubility measurement by the shake-flask method

The equilibrium solubility of the model drugs was measured by the harmonized protocol (HP), and the non-harmonized proprietary protocol of each company (nonHP). The protocol outlined below was presented to each company as the HP. To be viable at each company, the details of the HP were not fully specified. The solubility of the model drug in the JP 2^nd^ fluid at 37 °C was measured. The experiments were performed in triplicate.

1. Add an excess of a drug to a vial, then add a buffer solution (e.g. 5 mg/ 5 mL in a 15 mL vial).
2. After stirring for 1 min with a vortex mixer, shake the sample vigorously at 37 °C for 24 h with light shielding. If possible, measure the concentration-time profile^1^.
3. Let stand for a few minutes to sediment solids.
4. Filter the supernatant, discarding the first few portions (e.g., 0.1 mL for 4 mm diameter filter). Select an appropriate filter material. If possible, pre-heat the filter and syringe to 37 °C before use.
5. Dilute the filtrate as necessary. If possible, to avoid adsorption, pre-treat a tip with the filtrate, add a surfactant (a few microliters) to the filtrate, and/or rinse the tip with an organic solvent.
6. Measure the final pH.
7. Measure the concentration of a drug by an appropriate method (e.g. UV-Vis, HPLC/UV-Vis, or LC-MS).
8. If possible, analyse the initial and residual solids by powder X-ray diffraction (PXRD)^1^.

### Solubility measurement by μDISS Profiler

The solubility of the model drugs was also measured with a μDISS Profiler™ instrument (pION INC) by company F. This instrument employs eight fibre-optic dip probes, each with its own dedicated photodiode array spectrometer [39]. An excess of a model drug and 20 mL of a buffer solution pre-heated to 37 °C were added to the vessels and stirred at 37 °C for 24 h. The concentration of the model drug was measured by *in situ* UV probes (wave length 270-280 nm for albendazole, griseofulvin, dipyridamole, and 250-265 nm for glibenclamide). The experiments were performed in duplicate.

## Results and discussion

### Harmonizing the protocol for equilibrium solubility measurements by the shake-flask method

Consensus recommendations about the shake flask method have been summarized by Avdeef et al. [28]. However, since every company has its own situation, it may not be practical to pay equal attention to every recommendation. Therefore, we first divided the recommendations into two categories, “Mandatory” and “Recommended”, including a few additional recommendations based on their possible impact on solubility data. We then embodied these mandatory and recommended items as a harmonized protocol.

#### Mandatory

##### Agitation

Sufficient agitation should be applied to firmly wet and suspend drug particles. Poor wettability can often be a problem for poorly water-soluble drugs. Sonication is not recommended as it can promote aggregation of solids [28].

##### Incubation temperature

The temperature must be controlled. Solubility can vary depending on the drug, by a factor of 0.4 (e.g., erythromycin) to 6.5 (e.g., triflupromazine) between 25 °C and 37 °C [40].

##### Incubation time

The incubation time should be set to > 24 h to achieve equilibrium [28,41-43]. If possible, the concentration should be measured over time to ensure achieving equilibrium, for example at 1, 6, and 24 h [24].

##### Filtration

Lipophilic drugs tend to adsorb to the filter material [44,45,46]. A hydrophilic type filter (e.g., hydrophilic PVDF and PES) is recommended for unionized species [28]. The first few portions of a filtrate must be discarded to pre-saturate the filter.

##### Pipetting

Lipophilic drugs tend to adsorb to a pipet tip and vial materials [47,48]. To avoid adsorption, pipet tips can be pre-treated by the filtrate solution. Addition of a drop of surfactant is also effective for preventing adsorption. After pipetting, the tip can be rinsed with an organic solvent.

##### Final pH

The pH value can change during solubility measurements for ionizable drugs, especially when a large excess of an ionizable drug is added in a salt form.

##### Solid state characterization

The initial form may transform to a more stable form, a free form, a hydrate, etc. during solubility measurements. The solid forms of the initial solids and the residual solids after solubility measurements (equilibrium maker) should be characterized by PXRD, differential scanning calorimetry, Raman spectra, etc.

#### Recommended

##### Filtration temperature

To maintain the temperature of an equilibrated solution during filtration, it is recommended to pre-warm filters and syringes before filtration [49].

##### Light shielding

It is recommended to shade the sample during the solubility measurements.

##### Sedimentation after agitation

It is recommended to settle the residual solids after agitation to facilitate filtration. Sedimentation can also prevent the formation of a supersaturated solution by intensive stirring [28, 43].

The abovementioned mandatory and recommended items were embodied as a HP, considering practicability at each company (See experimental section). Some flexibility was left in the HP so that it could be practically applied to each company.

In the previous consensus recommendation paper, sedimentation was recommended as the first choice to separate solids from solution [28]. However, direct sampling from the supernatant requires some technical skills, especially when using small vials or microtubes. Fine powders may be re-suspended even by slight stimulation. Therefore, we used filtration in the harmonized protocol.

### Equilibrium solubility measured by nonHP and HP

**Figure 3.**
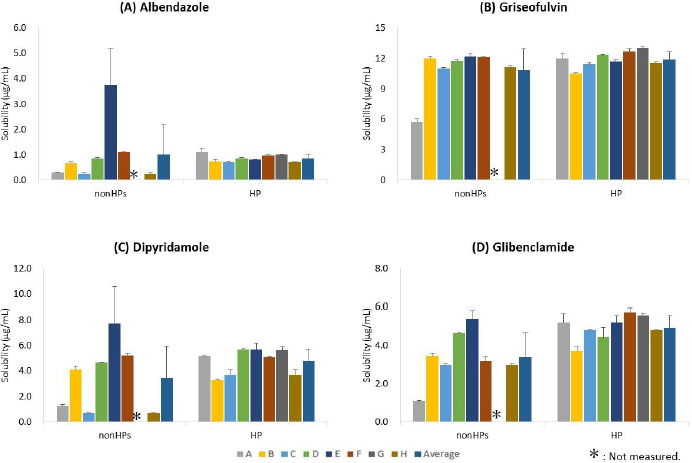
Solubility of model drugs measured by the non-harmonized proprietary protocols (nonHPs) and the harmonized protocol (HP).

Eight companies participated in this study (A to H). The equilibrium solubility of four model drugs in the JP 2^nd^ fluid (pH 6.8) was measured by the nonHP and HP (Tables 3 and 4, respectively). The comparisons of the solubility values between laboratories are shown in Figure 3.

The solubility values measured by the nonHPs showed marked inter-laboratory variance for albendazole, dipyridamole, and glibenclamide (CV = 113, 72, and 38 %, respectively). For griseofulvin, the inter-laboratory variance was smaller (CV = 20 %). The solubility of griseofulvin would be less susceptible to adsorption to a filter and plastic materials because its lipophilicity is lower than the other model drugs. The solubility values measured by A, C and H were lower than the others probably due to adsorption to filters and tips. On the other hand, the solubility measured by E was higher than the others probably because centrifugation was used to separate solids from a solution.

**Figure 4.**
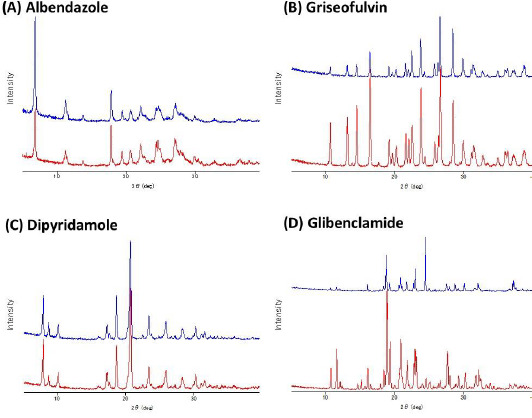
Powder X-ray diffraction patterns of samples obtained before (red lines) and after (blue lines) solubility assay (measured by company C) (Rigaku SmartLab diffractometer, Rigaku, Japan). The diffraction patterns were collected from 5° to 40° at a scan rate of 5°/min (Cu Kα radiation (40 kV and 30 mA), Rigaku D/teX ultra-highspeed position-sensitive detector).

In contrast to the nonHPs, the solubility values measured by the HP showed smaller inter-laboratory variance for all model drugs (CV < 20 %). Furthermore, the solubility values were in good agreement with the literature values (Table 5). The final pH values were within ± 0.03 of the initial pH values for all model drugs. The solid phases of the samples were evaluated by PXRD before and after solubility measurements (by A, B, C, F, and H). As shown in Figure 4, no solid form transformation occurred during the solubility measurements for all model drugs. The concentration-time profiles were measured to confirm achieving equilibrium by D, E, F, and G (data not shown).

**Figure 5:**
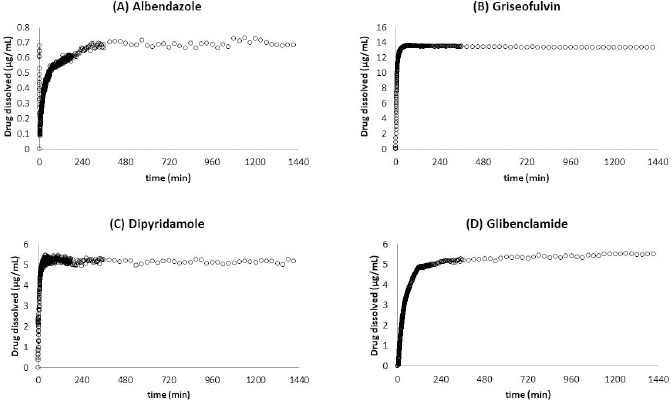
Dissolution profiles of model drugs by μDISS Profiler

**Figure 6:**
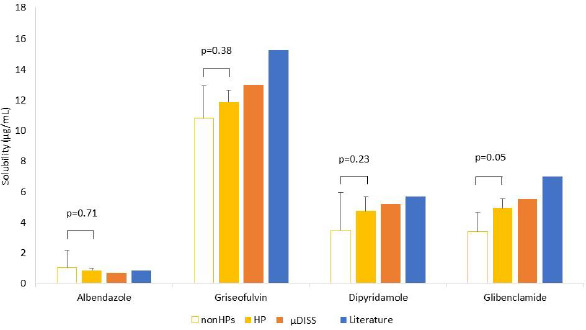
Comparison of equilibrium solubility at pH 6.8, 37 °C of model drugs obtained by different methods.

The solubility measurements were also performed by the μDISS Profiler. The dissolution profiles of the model drugs were shown in Figure 5. The solubility values of albendazole, griseofulvin, dipyridamole, and glibenclamide were 0.69, 13, 5.2, 5.5 μg/mL, respectively. These solubility values were similar to the values measured by the HP (Table 5 and Figure 6).

In this study, the pH value did not change during incubation, because the drug concentration was set to about 1 mg/mL (as of added solids) and the buffer capacity was set to be sufficient. However, in the case when a large amount of a drug and/or a buffer with limited buffer capacity are used, the final pH after incubation would be different from the initial pH. It should be noted that even when starting with a salt form, at the neutral pH region, the residual solids become a free form after achieving equilibrium if the pH is below (for acids) or above (for bases) the pH_max_ (the pH where the system changes from the pH-controlled region to the common-ion-effect-controlled region). Therefore, the observed solubility becomes the same regardless of the starting material being a salt or a free form if the solution is well-buffered to maintain the pH (unless there is a difference in the solid forms of residual free form solids). This point is often misunderstood by pharmacokinetic scientists who perform computational oral absorption simulation for a salt form drug substance. The dissolution of a salt and the supersaturation and phase separation of a free form must be taken into account to simulate the oral absorption of the drug. The *K*_sp_ (the drug-salt solubility product) value is at least required to simulate the dissolution of a salt. However, the dissolution mechanisms of a salt are not fully understood, especially the chemical reactions in the unstirred water layer [50, 51].

It is highly recommended to analyse the solid forms of both initial and residual solids. In drug discovery, little information about polymorphs and pseudo-polymorphs is available. Therefore, the information about the solid form obtained along with the solubility data is important for further solid form characterization.

For the assessment of drug solubility in physiological gastrointestinal conditions, an HCl solution of pH 1 to 2 is often used. In an HCl solution, the solubility of a drug is often controlled by the common ionic effect of the chloride ion. In this case, the residual solid after the solubility measurement becomes an HCl salt. In addition, attention should be paid to the formation of soluble drug-buffer complexes or insoluble drug-buffer precipitates in a phosphate buffer for basic drugs [52, 53]. The analysis of the residual solid after solubility measurements can clarify the formation of insoluble drug-buffer precipitates.

The harmonized protocol would be suitable for solubility measurements at the preclinical stages, e.g., the late lead optimization and pre-formulation stages. We successfully predicted the oral absorption of proprietary drug candidates in dogs using the solubility data measured by the HP [54]. It should be noted that the solid form may change during the preparation of a suspension formulation for a preclinical *in vivo* study.

## Conclusions

In conclusion, this study demonstrated that the inter-laboratory variance was successfully reduced by applying the harmonized solubility protocol. The harmonized protocol proposed in this study is practically applicable to drug discovery and development.

## Figures and Tables

**Table 1.** Summary of solubility measurement protocols of each company before harmonization.

Company	A	B	C	D	E	F	H
Vigorous agitation	yes	yes	no	yes	yes	yes	no
Temperature control	yes (25 °C)	yes (37 °C)	yes (37 °C)	yes (37 °C)	yes (37 °C)	yes (37 °C)	yes (37 °C)
Equilibration for > 24 h	no (18 h)	no (1 h)	yes (24 h)	yes (24 h)	yes (24 h)	yes (24 h)	yes (24 h)
Pre-saturation of filter	no	yes	no	yes	no ^a^	yes	no
Preventing adsorption	no	yes	no	yes	no	yes	no
Solid state characterization	no	no	no	yes	no	yes	no

^a^ Separated by centrifugation.

**Table 2.** Physicochemical properties of model drugs.

Compound	M_W_	p*K*_a_^a^	log *P*_oct_^b^	Lit. Solubility		Calculated solubility at pH6.8 ^c^, μg/mL	Ref.
				Solubility (37 °C), μg/mL	pH		
Albendazole	265	4.2 (base)	3.1	0.20 (25 °C)	7.4	0.20	31
		(25 °C)		0.95	7	0.95	32
				0.74	(Intrinsic solubility)	0.74	33
				0.85 ^d^	6.5	0.85	34
				1.1 ^d^	5	0.95	34
				0.80 ^d^	6.5	0.79	35
Griseofulvin	353	N/A^e^	2.5	5.3 (25 °C)	7.4	N/A	31
				15 ^d^	6.5	N/A	35
				15	6.5	N/A	36
				16	6.5	N/A	37
Dipyridamole	505	4.9 (base)	3.9	4.9	7	4.9	32
		(37 °C)		6.6 ^d^	6.5	6.5	35
				5.4	6.5	5.3	36
				6	6.5	5.9	37
Glibenclamide	494	5.2 (acid)	3.1	4.4 (25 °C)	7.4	1.1	31
		(37 °C)		5.6	7	3.6	32
				4.5 ^d^	6.5	8.8	34
				0.3 ^d^	5	7.5	34
				4.4 ^d^	6.5	8.7	35
				3.0	6.5	5.8	38
				0.30	5	7.5	38

^a^ Reference [29]

^b^ Reference [30]

^c^ Calculated by the Henderson-Hasselbalch equation from the reported solubility value, pH and pK_a_

^d^ Measured by μDISS Profiler

^e^ Not applicable

**Table 3.** Equilibrium solubility of model drugs in the JP 2^nd^ fluid (pH 6.8) obtained by the proprietary protocols of each company before harmonization.

Compound	Solubility (SD) (μg/ mL)								CV% ^a^
	A	B	C	D	E	F	H	Average	
Albendazole	0.29 (0.02)	0.67 (0.05)	0.25 (0.03)	0.86 (0.02)	3.74 (1.44)	1.10 (0.01)	0.25 (0.03)	1.02 (1.15)	113
Griseofulvin	5.7 (0.3)	12.0 (0.2)	11.0 (0.1)	11.7 (0.1)	12.2 (0.2)	12.1 (0.0)	11.1 (0.1)	10.8 (2.12)	20
Dipyridamole	1.24 (0.11)	4.08 (0.29)	0.66 (0.02)	4.63 (0.02)	7.69 (2.88)	5.22 (0.14)	0.66 (0.02)	3.45 (2.49)	72
Glibenclamide	1.1 (0.0)	3.4 (0.1)	3.0 (0.0)	4.6 (0.0)	5.4 (0.4)	3.2 (0.2)	3.0 (0.0)	3.4 (1.3)	38

^a^ Coefficient of variation of solubility values obtained from 7 companies.

**Table 4.** Equilibrium solubility of model drugs in the JP 2^nd^ fluid (pH 6.8) obtained by the harmonized protocol.

Compound	Solubility (SD) (μg/ mL)									CV% ^a^
	A	B	C	D	E	F	G	H	Average	
Albendazole	1.11 (0.14)	0.73 (0.08)	0.71 (0.03)	0.86 (0.02)	0.79 (0.02)	0.96 (0.04)	1.01 (0.02)	0.70 (0.03)	0.86 (0.14)	17
Griseofulvin	12.0 (0.5)	10.5 (0.1)	11.4 (0.2)	12.3 (0.1)	11.7 (0.1)	12.7 (0.3)	13.0 (0.1)	11.5 (0.2)	11.9 (0.75)	6
Dipyridamole	5.15 (0.02)	3.27 (0.06)	3.69 (0.37)	5.65 (0.09)	5.67 (0.50)	5.07 (0.09)	5.62 (0.25)	3.70 (0.37)	4.73 (0.94)	20
Glibenclamide	5.2 (0.4)	3.7 (0.2)	4.8 (0.0)	4.4 (0.5)	5.2 (0.3)	5.7 (0.2)	5.6 (0.1)	4.8 (0.0)	4.9 (0.6)	13

^a^ Coefficient of variation of solubility values obtained from 8 companies.

**Table 5.** Average values of equilibrium solubility of model drugs obtained by different methods at pH 6.8, 37 °C.

Compound	Solubility							
	(μg/mL)				(log, M)			
	nonHPs	HP	μDISS	Literature	nonHPs	HP	μDISS	Literature
Albendazole	1.02	0.86	0.69	0.86 ^a^	-5.41	-5.49	-5.58	-5.49
Griseofulvin	11	12	13	15 ^b^	-4.51	-4.47	-4.43	-4.36
Dipyridamole	3.5	4.7	5.2	5.7 ^c^	-5.17	-5.03	-4.99	-4.95
Glibenclamide	3.4	4.9	5.5	7.0 ^d^	-5.16	-5.00	-4.95	-4.85

^a^ Reference [32-35]

^b^ Reference [35-37]

^c^ Reference [35-37]

^d^ Reference [32, 34, 35, 38]
